# Salivary Antioxidants Status Following Progressive Aerobic Exercise: What Are the Differences between Waterpipe Smokers and Non-Smokers?

**DOI:** 10.3390/antiox8100418

**Published:** 2019-09-20

**Authors:** Hamid Arazi, Behzad Taati, Forough Rafati Sajedi, Katsuhiko Suzuki

**Affiliations:** 1 Department of Exercise Physiology, Faculty of Sports Sciences, University of Guilan, Rasht 4199843653, Iran; taati.behzad@yahoo.com; 2 Department of Sport Sciences, Faculty of Humanities Sciences, Islamic Azad University, Rasht Branch, Rasht 4147654919, Iran; frafatisajedi@yahoo.com; 3 Faculty of Sport Sciences, Waseda University, Tokorozawa 359-1192, Japan; katsu.suzu@waseda.jp

**Keywords:** uric acid, peroxidase, DPPH, saliva, hookah, free radicals

## Abstract

Waterpipe tobacco (WPT) smoking is a public health problem with similar or even stronger effects than cigarette smoking. Although it appears to be associated with extensive oxidative stress, there is a limited number of studies on the oxidative effects of WPT smoking in stressful conditions. We, therefore, compared the responses of salivary flow rate (SFR), uric acid (UA) concentration, and peroxidase (POX) and 2,2-diphenyl-1-picryl-hydrazyl-hydrate (DPPH) activities between WPT smokers and non-smokers following a bout of exhaustive aerobic exercise (AE). Twenty-three sedentary young women (age: 22.95 ± 2.83 years) participated in this study, including 11 smokers (7.00 ± 1.41 uses/week) and 12 non-smokers. All participants were required to perform the Bruce protocol treadmill test at an initial gradient of 10% at 1.7 mph, with increases of these parameters every 3 min until exhaustion. Unstimulated saliva samples were collected before, immediately after, and 1 hour after AE. WPT smokers showed lower SFR compared with non-smokers at all time points (*p* < 0.05). In comparison to WPT smokers, a larger increase in POX activity (approximately 23% vs. 14%; *p* = 0.009) and a smaller decline in DPPH activity (approximately −8% vs. −15%; *p* = 0.004) were found in non-smokers compared with WPT smokers. While these changes were slowly compensated within 1 hour after exhaustion, the activity of both markers was different from the pre-exercise values (*p* < 0.001). There was also a trend for UA concentration in non-smokers to be higher during the recovery period, with no significant difference between the groups (*p* > 0.05). It seems that WPT smoking is associated with negative effects on important human antioxidants and a diminished antioxidative response following acute exercise.

## 1. Introduction

Tobacco smoking is one of the most common leading causes of death in the world because it is associated with many types of non-communicable chronic disease such as cancers, cardiovascular diseases, and respiratory problems [1,2]. It has been suggested that tobacco smoking induces these hazardous health effects through the production of free radicals in users [3]. Although contracting skeletal muscles continuously generate reactive oxygen species (ROS) whose presence is obligatory for normal physiological functions [4], tobacco smoke contains large amounts of ROS, which randomly react with various molecules including lipids, DNA, and proteins [5,6]. This condition known as oxidative stress (OS) plays a critical role in initiating local and systemic inflammation after smoking [7]. Taken together, increased OS and deterioration of the antioxidant defense system are the main mechanisms to explain the harmful effects of inhaled smoke [8,9].

Cigarette smoking and waterpipe tobacco (WPT) or hookah smoking are two major forms of tobacco consumption by the adult population [2], with Iranian women more likely to smoke hookah and showing a growing tendency towards WPT smoking [10,11]. The popularity of hookah smoking may be due to the common misperception that the water used in the bowl of hookah filters the smoke and therefore WPT smoking is less harmful than cigarette smoke [3]. However, WPT smoking produces more smoke, greater carbon monoxide, and similar nicotine when compared with cigarette smoking [12]. Moreover, there is overwhelming evidence that the impact of WPT smoke on human antioxidant status may be similar or even worse than that of cigarette smoking [9].

On the other hand, although regular exercise training increases the activity of DNA repair enzymes involved in ROS-mediated damage [13], acute physical exercise can induce different degrees of OS, depending on the intensity, duration, and type of exercise [14]. We previously found that a bout of exhaustive aerobic exercise (AE) increases salivary antioxidant factors including peroxidase (POX) activity and uric acid (UA) concentration and decreases 2,2-diphenyl-1-picryl-hydrazyl-hydrate (DPPH) activity up to 1 h following exercise in both smoker and nonsmoker young women [15]. It was also reported that salivary flow rate (FR) in smokers is lower than in non-smokers [16]. Considering that, as observed for cigarette smoking, hookah smoke exposure elevated OS markers in animal models [3,17] and its detrimental effects on the antioxidant defense system could be even worse than those of cigarette smoke [9], this study was designed to investigate the responses of salivary FR, POX activity, UA concentration, as well as DPPH activity after exhaustive AE in WPT smoker women in comparison with non-smokers. To the best of our knowledge, no data are available concerning the acute antioxidative responses of WPT smokers to an extreme physical exercise session. 

## 2. Materials and Methods

### 2.1. Participants

**Figure 1:**
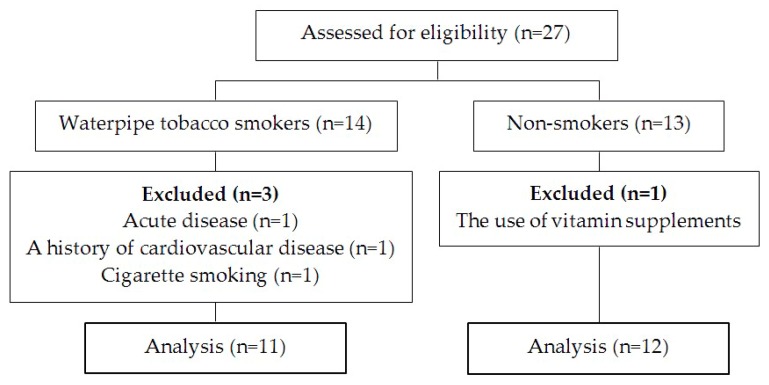
Flow chart of the study from assessment of eligibility to data analysis.

Twenty-three young women, including 11 WPT users (5 to 9 uses per week) and 12 non-users, were selected from a total of 27 volunteers according to the entry criteria, i.e., no regular exercise training in the last 3 months, no current pregnancy, lack of any oral infection and acute disease, and no signs, symptoms, or history of cardiovascular, respiratory, and musculoskeletal diseases. All participants did not use drugs in the past month and supplements known to affect the results (i.e., vitamin supplements) in the past six months. WPT smokers also did not smoke tobacco products other than hookah (Figure 1). 

The demographic characteristics of participants are shown in Table 1. Each participant signed an informed consent for inclusion after receiving an extensive verbal and written description of the study. This study was conducted in accordance with the Declaration of Helsinki, and the protocol was approved by the Ethics Committee of Islamic Azad University (IAU1396620). 

### 2.2. Study Procedures

A familiarization session was considered one week before the test day to familiarize participants with the AE protocol and collection of saliva samples. Descriptive characteristics of each participant including height, weight, and body fat percentage were also measured at the end of this session. Participants were instructed to maintain their current dietary routines during the following week. However, to control energy intake, each participant received both written and verbal instructions to record the type and portion sizes of her daily food (two working days and one weekend day). 

Participants were instructed to eat their last meal at 11 am along with 500 mL of water to reach the same hydration level and refer to the gym at 2 pm. After arriving, they brushed their teeth and rinsed their mouth with distilled water. A list of foods with high antioxidant properties (such as tomato, tea, garlic, etc.) was provided to all participants, and they were not permitted to use any of these foods at least 24 h before the exercise test. Pretest saliva samples were collected before the exercise during the final 5 min of a 15 min rest on a comfortable chair. The second and third whole saliva samples were also obtained immediately and 1 h after the exercise, respectively. Participants did not eat or drink between the samplings. WPT smokers were also instructed to avoid smoking on the day before the testing. It should be noted that all the study procedures including data collection and familiarization were time-matched between the groups and performed in a temperature-controlled room (22–24 °C) to avoid circadian variations in salivary antioxidant status and exercise performance.

### 2.3. Exercise Protocol 

Initially, all participants completed a 10 min warm-up period including 5 min walking on a treadmill with no gradient followed by lower limb stretching exercises. The graded AE protocol was according to the Bruce protocol treadmill test. The test started by walking at 1.7 mph (2.74 km/h) at a gradient of 10% for the first 3 min. Because the exercise workload was increased gradually, the AE protocol proceeded with running during next stages. The gradient of the treadmill was increased by 2% every 3 min, and the speed was 2.5, 3.4, 4.2, 5, 5.5, and 6 mph in the subsequent six stages [18]. The protocol was performed until the participant could not continue the exercise (exhaustion). Participants were encouraged to complete the AE protocol as long as possible. Rating of perceived exertion (RPE) and heart rate (HR) were also recorded at the end of every stage using Borg’s RPE 6–20 scale and an automatic HR monitor (Beurer, PM80, Ulm, Germany), respectively.

### 2.4. Saliva Collection and Determination of Flow Rate

Saliva is the first biological fluid containing different antioxidant molecules and enzymes that protect the body cells against free radical oxidation [15]. Therefore, the collection of saliva to compare the antioxidant status in smokers and non-smokers could be informative. Participants collected their un-stimulated saliva in dry and sterile tubes (1.5 mL). The time required to collect 1 ml of saliva was considered as FR [16,19]. The samples were then centrifuged at 3000 rpm for 10 min and stored at −30 °C for further analyses. 

### 2.5. Salivary POX Activity

POX activity was determined using 4-amino antipyrine in the presence of hydrogen peroxide [20]. A 0.3 M phosphate buffer (3 mL, pH = 7.4) containing 0.0010 M hydrogen peroxide, 0.002 M 4-amino antipyrine, and 0.15 M phenol was used to measure the oxidation of 4-amino antipyrine at 25 °C. A 40 mL volume of enzyme solution (40 µL, 6 ×10^−4^ mg/mL in 0.3 M phosphate buffer, pH 7.4) was then added, and alterations in absorption were recorded at 510 nm (ΔA/min). One unit of enzyme activity was defined as an absorbance change of 0.001 per min under standard conditions [15,21].

### 2.6. Salivary UA Assay

Salivary UA concentrations were measured using an enzymatic method that was previously described by Trivedi et al. [22]. The enzymatic reaction of uricase with UA and the formation of 5-ureidohydantoin (allantoin) and hydrogen peroxide (H_2_O_2_) coupled with the catalytic oxidation of *p*-hydroxybenzoate and 4-aminoantipyrine in the presence of POX were the basis of UA concentration assessment. The produced pink chromophore was detected at 505 nm.

### 2.7. DPPH Radical Scavenging Assay

A spectrophotometer (Ultrospec 3000 UV/Vis, Pharmacia Biotech, Piscataway, USA) was utilized to assay the radical scavenging activity of the samples against stable DPPH, on the basis of a method that was previously described by Bompadre et al. [23]. A UV/visible light spectrophotometer measured alterations in DPPH color from deep violet to light yellow at 517 nm. A fresh methanolic solution of DPPH (1500 µL) and 77 µL of centrifuged saliva were mixed and kept in the dark for 30 min at room temperature, and the absorption was then determined at 517 nm using methanol as a blank. The absorbance of a methanolic solution of DPPH was also considered as a control value. All procedures were carried out in duplicate, and the radical scavenging activity was calculated using the following equation [15,16]:

DPPH radical scavenging activity (%) = [(Ac − AS)/Ac] × 100

### 2.8. Data Analysis

A priori power analysis using G*Power software, version 3.1.9.4 (Franz Faul, Christian-Albrechts-Universität Kiel, Kiel, Germany) [24], with an α level of 0.05 and a statistical power of 0.90 was performed to detect the sample size required for the study. Data analysis was performed using the SPSS software (version 18^®^; SPSS Inc, Chicago, IL, USA), and a *p* value less than 0.05 was considered significant. Prior to any analysis, the Shapiro–Wilk test was used to verify data normality. The statistical analysis of dietary intake was accomplished using independent samples *t*-tests. Statistical significance was assayed by application of repeated measures ANOVA with 2 conditions (WPT smokers or non-smokers) × 3 times (pre, immediately, and 1 h after exercise). The sphericity assumption was met, and post-hoc comparisons were made by the Bonferroni test when necessary.

## 3. Results

Dietary data were analyzed by the *Nutritionist IV diet analysis software* (1995, First Databank, San Bruno, CA, USA). Table 2 shows the approximate amount of energy, carbohydrate, protein, fat, and vitamin E and C in both groups. There were no significant differences between smokers and non-smokers (*p* > 0.05).

HR and RPE were recorded at the end of each stage throughout the exercise protocol to monitor the rate of fatigue and the ability to continue the exercise on the treadmill. These parameters are shown in Table 3.

**Figure 2:**
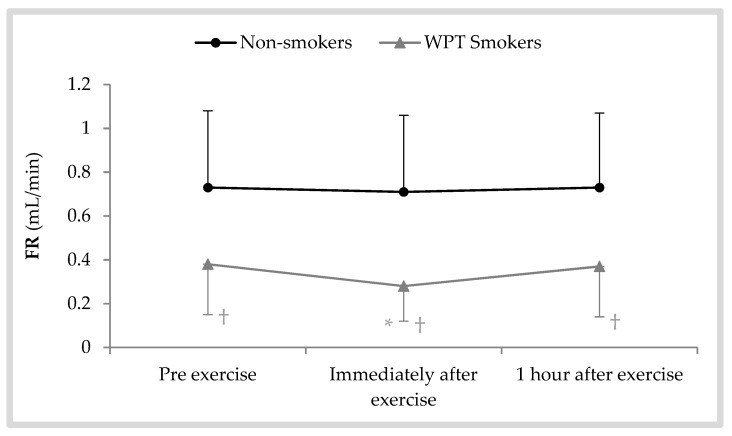
Saliva flow rate (FR) following a bout of exhaustive aerobic exercise for WPT smokers and non-smokers; * *p* < 0.05 vs. pre, ^†^ *p* < 0.05 vs. non-smokers.

The changes in salivary flow rate during the 1 h recovery period after exhaustive AE are shown in Figure 2. There was a significant effect of the time of sample collection [F (2, 1.06) = 4.65; *p* = 0.040; η^2^ = 0.18]. In WPT smokers, the flow rate immediately after AE was significantly lower than before exercise (mean diff = −0.097 ± 0.11; *p* < 0.001). However, the changes in non-smokers did not indicate a time effect (*p* = 0.068). Between-group comparisons revealed that the pre-exercise differences between WPT smokers and non-smokers (*p* = 0.013) as well as the differences immediately (*p* = 0.028) and 1 h (*p* = 0.014) after exercise were significant statistically. 

**Figure 3:**
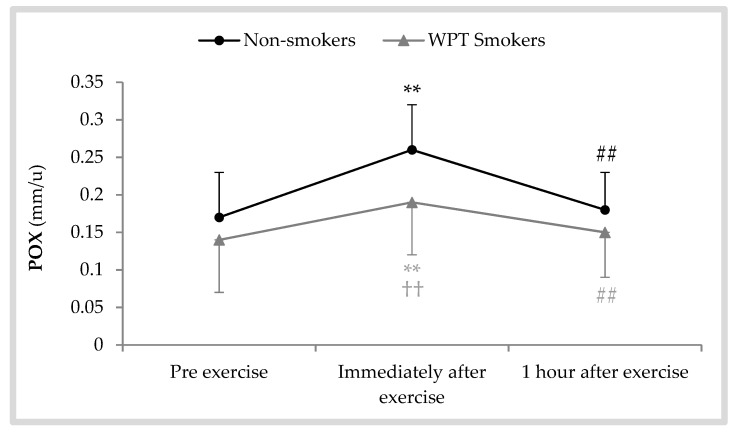
Salivary peroxidase (POX) activity following a bout of exhaustive aerobic exercise in WPT smokers and non-smokers; ** *p* < 0.01 vs. pre, ^##^ *p* < 0.01 vs. immediately after, ^††^ *p* < 0.01 vs. non-smokers.

Alterations in salivary POX activity following exhaustive AE are shown in Figure 3. Repeated-measures ANOVA revealed a significant effect of the collection time [F (2, 42) = 78.88; *p* < 0.001; η^2^ = 0.79]. On the basis of pairwise comparisons using the Bonferroni test, POX activity immediately after exercise was higher than before exercise in both WPT smokers (mean diff = +0.18 ± 0.06; *p* < 0.001) and non-smokers (mean diff = +0.12 ± 0.06; *p* < 0.001). These values then decreased near to pre-exercise levels, as POX activity 1 h after exercise was lower than immediately after exercise in both WPT smokers (mean diff = −0.09 ± 0.03; *p* < 0.001) and non-smokers (mean diff = −0.14 ± 0.07; *p* < 0.001). Moreover, in between-group comparisons, no statistically significant differences were found before exercise (*p* = 0.142) and 1 h after that (*p* = 0.085), whereas a significant difference was only observed immediately after exercise (*p* = 0.009). 

**Figure 4:**
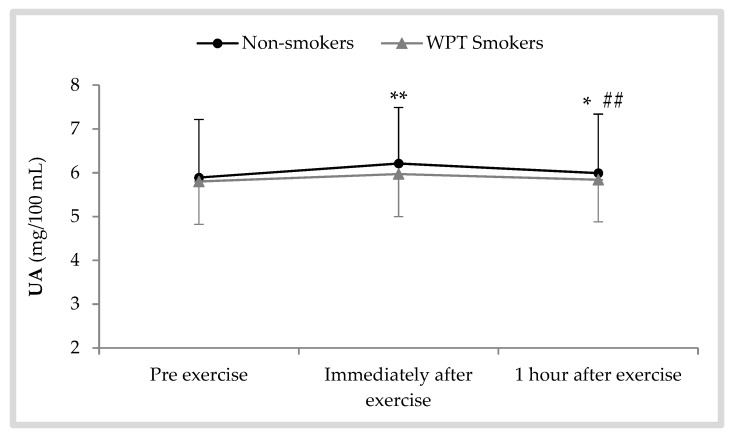
Salivary uric acid (UA) concentrations following a bout of exhaustive aerobic exercise in WPT smokers and non-smokers; * *p* < 0.05 vs. pre, ** *p* < 0.01 vs. pre, ^##^ *p* < 0.01 vs. immediately after.

In relation to salivary UA changes indicated in Figure 4, there was a significant effect of the collection time [F (1.54, 34.03) = 23.39; *p* < 0.001; η^2^ = 0.51]. However, no significant differences were found between WPT smokers and non-smokers before exercise (*p* = 0.854), immediately after exercise (*p* = 0.615), or 1 h after exercise (*p* = 0.771). Although the mean concentration of UA was higher immediately and 1 h after AE in both groups compared to pre-exercise levels, these changes were only significant in non-smokers (immediately after exercise: + 0.32 ± 0.20; *p* = 0.001, 1 h after exercise: + 0.10 ± 0.09; *p* = 0.012).

**Figure 5:**
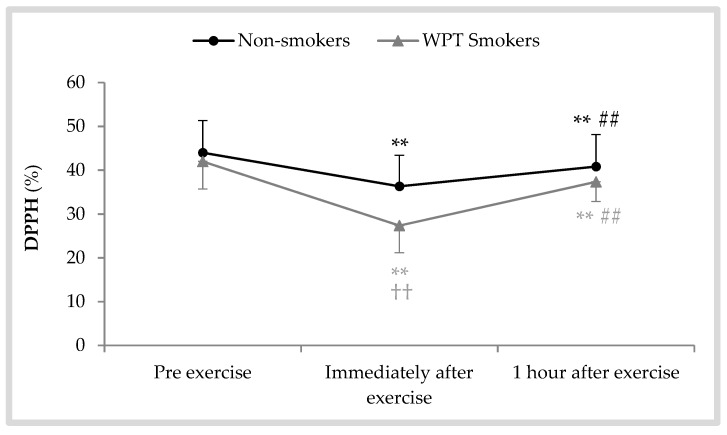
Salivary 2,2-diphenyl-1-picryl-hydrazyl-hydrate (DPPH) activity following a bout of exhaustive aerobic exercise in WPT smokers and non-smokers; ** *p* < 0.01 vs. pre, ^##^ *p* < 0.01 vs. immediately after, ^††^ *p* < 0.01 vs. non-smokers.

As shown in Figure 5, a significant effect of the collection time existed for the percentage of DPPH radical scavenging activity [F (1.37, 30.21) = 71.22; *p* < 0.001; η^2^ = 0.76]. DPPH activity was decreased significantly in both WPT smokers (immediately after exercise: −14.63 ± 5.20; *p* < 0.001, 1 h after exercise: −4.63 ± 3.26; *p* = 0.002) and non-smokers (immediately after exercise: −7.66 ± 2.38; *p* < 0.001, 1 h after exercise: −3.16 ± 1.52; *p* < 0.001). However, the observed differences between the groups were found to be statistically significant only immediately after exercise (*p* = 0.004). 

## 4. Discussion

As it has been well documented in previous research [14,15,19,21], the present results also proved that acute strenuous AE induces OS in young people. Increased free radicals in this situation, specifically ROS, may cause serious damages to cells [25]. Thus, antioxidant defense system plays a critical role in protecting the cells against free radicals produced by stressful conditions. In this context, we previously observed that cigarette smoking prevents suitable antioxidant responses after exhaustive exercise in smoker young women [15]. Although the negative effects of other forms of tobacco consumption such as WPT smoking may be similar to or even stronger than those of cigarette smoking, little attention has been paid to the long-term effects of WPT smoking on the human antioxidant defense system. Therefore, in the present study, the response of some important salivary antioxidant markers following a bout of exhaustive AE was measured in a group of WPT smoker young women and compared with that of non-smokers. 

We observed that saliva FR in WPT smokers before exercise and immediately and 1 h after an exhaustive AE was lower than in non-smoker women. These findings confirm that the presence of various toxic chemicals in hookah smoke may decrease saliva FR in smokers [16]. As observed in the present study, Damirchi et al. [21] reported that the salivary FR was not significantly altered by increased exercise intensity in non-smoker healthy men. The same results were also obtained in another study, and it was concluded that dehydration during an intense AE does not seriously affect the normal FR of saliva [19].

The results obtained in the present study revealed that the responses of salivary POX activity and DPPH radical scavenging activity in WPT smokers were weaker than in non-smokers, particularly immediately after exercise. It was also observed that the responses of salivary UA could differ between WPT smokers and non-smokers during the recovery period after an exhaustive AE. In comparison to WPT smokers, non-smokers experienced a larger increase in POX activity (around 23% vs. 14%) and a smaller reduction in DPPH activity (around −8% vs. −15%) immediately after exercise. Moreover, UA concentrations in non-smokers tended to be higher than in WPT smokers following exhaustive AE, but the differences were not significant. 

Enzymatic and non-enzymatic antioxidants protect the body against the adverse effects of OS. Overall, the antioxidant capacity is determined by these antioxidative compounds [26]. The salivary antioxidative defense system contains a variety of molecules and enzymes. As one of the most important antioxidants, UA accounts for approximately 70% of the total antioxidant capacity (TAC) of saliva [27], and an increase in UA concentration can improve TAC [14]. Furthermore, salivary POX, a pivotal enzyme in the oral antioxidative system, acts as a catalase to inhibit the accumulation of toxic levels of H_2_O_2_ that are produced after acute physical exercise [21,28]. Thus, it seems that the functions of both UA and POX are critical for the efficacy of the salivary antioxidative defense system. However, it has been found that TAC is significantly lower in WPT smokers than in non-smokers [29]. Likewise, an animal study involving a one-month nose-only exposure to WPT smoke showed that the level and activity of antioxidative markers were significantly decreased after the exposure period [17]. The impaired oxidant defense system observed in the present study confirms the above findings.

It is well established that extreme physical exercise is associated with high free radicals production, including that of superoxide, hydrogen peroxide, and hydroxyl radical, as a result of high oxygen consumption [30]. In the present study, increased salivary UA concentration and POX activity in non-smokers following the exercise protocol can be a marker of increased levels of free radicals. However, the mean responses in WPT smokers were lower due to their impaired antioxidative system. Our data indicate that excessive production of free radicals and metabolic substances (probably H_2_O_2_ and ROS) as a result of increasing exercise intensity to exhaustion may induce a vigorous stress on the salivary antioxidant defense system, specifically in WPT smokers, because UA concentrations were significantly increased only in non-smokers, and POX activity in non-smokers after exercise were significantly higher than in WPT smokers. These findings are in line with those of our previous experimental study [15] that revealed that the responses of the salivary antioxidant defense system following an exhaustive cycling exercise were less efficient in a group of cigarette smokers compared with a group of non-smokers. Thus, WPT smoking is associated with an antioxidative dysfunction similar to that seen following cigarette smoking. 

In our experiment, DPPH analysis was used to assess peroxy radical clearance based on available studies [15,31]. DPPH measurement in saliva is an easy, simple, and reproducible method to assay potential antioxidants [31,32]. As a stable solid radical source, DPPH can absorb one equivalent of electrons or hydrogen atom at ambient temperature [33]. Therefore, it is considered a well-known trap or scavenger for other radicals. In this study, the rate of DPPH activity following exhaustive AE decreased significantly in both groups. However, the percent of decline in WPT smokers was approximatly 7% higher than in non-smokers. Former research conducted on cigarette smokers has also reported similar observations [15,16]. For example, Nosratabadi et al. [16] compared the salivary antioxidant activity of healthy smokers with that of non-smokers in a similar age range. The authors demonstrated that the activity of saliva against DPPH radical in smokers was approximatly 25% lower than in non-smokers. Available data relating to DPPH activity support the idea that tobacco smokers are exposed to higher OS following acute exercise to exhaustion.

## 5. Conclusions

In conclusion, the present results allow us to assert that a bout of exhaustive AE on a treadmill induces OS in both non-smoker and WPT smoker young women. However, it seems that the responses of salivary antioxidative markers including POX activity and the percent of scavenging activity against DPPH radical after the exercise were weaker in WPT smokers. Therefore, contrary to popular belief, these findings provide experimental evidence that WPT smoking presents health risks similar to those of cigarette smoking. Further research in this field is needed to compare the effects of different forms of tobacco smoking on the responses of human antioxidants following acute exercise.

## Figures and Tables

**Table 1:** Descriptive characteristics of waterpipe tobacco (WPT) smoker and non-smoker young women.

Variable	WPT Smokers (*n* = 11)	Non-Smokers (*n* = 12)
**Age** (years)	23.63 (2.90)	22.66 (2.90)
**Height** (cm)	166.81 (4.99)	167.75 (5.84)
**Weight** (kg)	66.54 (8.45)	64.50 (7.74)
**Body mass index** (kg/m^2^)	22.19 (2.94)	23.17 (2.40)
**Body fat** (%)	29.72 (4.77)	27.25 (4.26)
**VO_2max_** (mL kg^−1^ min^−1^)	25.81 (4.66)	28.75 (4.13)
**Rate of smoking** (number/week)	7.00 (1.41)	-
**Time to exhaustion** (min)	7.07 (1.25)	8.09 (1.12)

Data are presented as mean (SD).

**Table 2:** Dietary information during the week prior to testing of WPT smoker and non-smoker young women.

Variable	WPT Smokers	Non-Smokers	*p*
**Energy intake** (Cal)	2482.30 (252.57)	2588.71 (244.93)	0.317
**Carbohydrate** (g)	373.49 (31.85)	406.45 (36.17)	0.098
**Protein** (g)	99.42 (17.36)	110.50 (16.38)	0.107
**Fat** (g)	75.58 (6.39)	81.13 (5.37)	0.089
**Vitamin E** (mg)	16.7 (3.21)	15.90 (4.80)	0.804
**Vitamin C** (mg)	77.52 (8.44)	74.29 (6.53)	0.683

Data are presented as mean (SD).

**Table 3:** Heart rate (HR) and rating of perceived exertion (RPE) for WPT smoker and non-smoker young women.

Variable	The Entire Period of Exercise		Before the End of Exercise	
	WPT Smokers	Non-Smokers	WPT Smokers	Non-Smokers
**HR** (bpm)	158.30 (9.60)	153.40 (8.70)	193.20 (7.90)	189.50 (6.20)
**RPE** (score)	13.50 (0.80)	14.60 (0.70)	19.10 (0.40)	18.80 (0.70)

Data are presented as mean (SD).
